# PEG Graft Polymer Carriers of Antioxidants: In Vitro Evaluation for Transdermal Delivery

**DOI:** 10.3390/pharmaceutics12121178

**Published:** 2020-12-03

**Authors:** Justyna Odrobińska, Magdalena Skonieczna, Dorota Neugebauer

**Affiliations:** 1 Department of Physical Chemistry and Technology of Polymers, Faculty of Chemistry, Silesian University of Technology, 44-100 Gliwice, Poland; justyna.odrobinska@polsl.pl; 2 Department of Systems Biology and Engineering, Silesian University of Technology, Akademicka 16, 44-100 Gliwice, Poland; 3 Biotechnology Centre, Silesian University of Technology, Krzywoustego 8, 44-100 Gliwice, Poland

**Keywords:** micellar carriers, conjugates, graft copolymers, cytotoxicity, artificial skin permeation test, cosmetology

## Abstract

The in vitro biochemical evaluation of the applicability of polymers carrying active substances (micelles and conjugates) was carried out. Previously designed amphiphilic graft copolymers with retinol or 4-*n*-butylresorcinol functionalized polymethacrylate backbone and poly(ethylene glycol) (PEG) side chains that included Janus-type heterografted copolymers containing both PEG and poly(ε-caprolactone) (PCL) side chains were applied as micellar carriers. The polymer self-assemblies were convenient to encapsulate arbutin (ARB) as the selected active substances. Moreover, the conjugates of PEG graft copolymers with ferulic acid (FA) or lipoic acid (LA) were also investigated. The permeability of released active substances through a membrane mimicking skin was evaluated by conducting transdermal tests in Franz diffusion cells. The biological response to new carriers with active substances was tested across cell lines, including normal human dermal fibroblasts (NHDF), human epidermal keratinocyte (HaCaT), as well as cancer melanoma (Me45) and metastatic human melanoma (451-Lu), for comparison. These polymer systems were safe and non-cytotoxic at the tested concentrations for healthy skin cell lines according to the MTT test. Cytometric evaluation of cell cycles as well as cell death defined by Annexin-V apoptosis assays and senescence tests showed no significant changes under action of the delivery systems, as compared to the control cells. In vitro tests confirmed the biochemical potential of these antioxidant carriers as beneficial components in cosmetic products, especially applied in the form of masks and eye pads.

## 1. Introduction

Carriers of active substances are a topic often examined due to the high demand for medical and cosmetic products that ensure a controlled course of delivery and release of substances. Many biologically active substances are unstable and sensitive to temperature, pH, light, and oxidation; therefore, they require carriers that protect them against undesirable degradation [1]. Desirable features of carriers used in medicine or cosmetology include their biocompatibility, non-immunogenicity, non-toxicity, and lack of accumulation within the body [2,3]. Polymeric drug delivery systems, such as micelles and polymer-drug conjugates, are often tested as controlled release carriers, due to their potential preparation from biodegradable, non-toxic polymers [1], as well as their synthesis via stimuli sensitive copolymers [4,5,6]. In dermatology and cosmetology, the new carriers and bioactive systems with potential transdermal therapy need to be extensively tested in vitro, before admission into the next phase of in vivo biological research.

An important aspect for the determination of newly prepared transdermal systems is the ability to penetrate and diffuse through the skin and/or diffusion of the released active substance, including drug. In vitro tests of absorption through the skin have been applied for parabens [7], ketoprofen released by a gel formulation and a conventional suspension [8], timolol maleate for glaucoma through the cornea [9], pirfenidone in idiopathic pulmonary fibrosis [10], lidocaine released by liposomes or lipid–polymer hybrid nanoparticles [11].

Depending on the target application, the cytotoxicity may be desirable activity (cancer therapy) or is a feature that causes disqualification of the bioactive substance/carrier (cosmetics) [12]. Comprehensive knowledge on type of cell death, including apoptosis [13], and cell cycle analysis [14] is standard biological assessment of polymer conjugates and micelles carrying active substances beneficial in cosmetology or medicine. No cytotoxicity of various polymethacrylates, i.e., poly(ionic liquid)s with salicylate or sulfacetamide anions [15,16], star-shaped conjugates with fluorescein or doxorubicin [17,18,19], and antipsoriatic prodrugs [20] has been reported when they were exposed to normal human dermal fibroblasts (NHDF). In the case of retinoids applicable in cosmetology, the non-toxicity of carriers, e.g., solid lipid nanoparticles [21] or phosphorylcholine prodrug liposomal formulation [22], has been also described.

We focused on the biological evaluation of polymer delivery systems of active substances in cosmetology. As we reported earlier, novel polymeric carriers based on amphiphilic polymers were synthesized by a controlled radical polymerization and “click” chemistry reaction, to afford PEG grafted and PEG/PCL co-grafted polymethacrylates [23,24]. These polymers showed the ability to self-assemble into micellar structures and encapsulate substances, such as arbutin (ARB). PEG graft polymers were conjugated with ferulic acid (FA) and lipoic acid (LA) [25]. An additional benefit of these polymers is the presence of retinol or 4-*n*-butylresorcinol as the terminal or center unit in the main chain, as it was also postulated for linear polymethacrylates [26,27]. Previous studies on the physicochemical properties and release kinetics have appeared to be attractive for their potential application in the skin treatment. Thus, these new polymethacrylate carriers required biological evaluation as the supplementary characteristics, to confirm their safe activity in the dermal application. The permeability of the active substance release through a membrane mimicking the skin was tested by transdermal tests in Franz diffusion cells. Due to the unique construction of the Franz cells, it is possible to monitor the movement of the substance from the donor chamber (surrounding area) through the membrane as the artificial skin to the acceptor chamber (organism). Moreover, the response of these new polymer systems was assessed by their exposure to the selected cell lines, including normal human dermal fibroblasts (NHDF), and human epidermal keratinocyte (HaCaT), as well as cancer melanoma (Me45), and metastatic the human melanoma cell line (451-Lu) as the problematic skin. The results were achieved by the use of cell cycle test as well as MTT, Annexin-V apoptosis assays, and senescence tests, which determine the occurrence and type of cell death.

## 2. Materials and Methods

### 2.1. Materials

Sodium phosphate buffer saline (PBS, 0.1 M, pH = 7.4), 3-(4,5-dimethylthiazol-2-yl)-2,5-diphenyltetrazolium bromide (MTT), DMEM-F12 medium, senescence cells histochemical staining kit, and trypsin were received from Aldrich (Poznań, Poland). Hydrochloric acid (HCl, 35–38%) and 2-propanol (98%) were received from Chempur (Piekary Śląskie, Poland). Physiological saline without Ca&Mg (PBS, PAA, Poland), Annexin-V apoptosis assay (BioLegend, San Diego, CA, USA), Annexin V Binding Buffer (BD Biosciences, San Jose, CA, USA), and propidium iodide solution (BD Biosciences, San Jose, CA, USA) were used as received. Normal human dermal fibroblasts (NHDF) were obtained from Lonza (Lonza; Celllab, Warsaw, Poland). Malignant melanoma cells (Me45) and a human metastatic melanoma variant of the WM164 cell line (451-Lu) were obtained from the collection of the Maria Sklodowska-Curie Memorial Cancer Center and Institute of Oncology (Gliwice, Poland). Spontaneously immortalized human epidermal keratinocytes (HaCaT) were purchased from CSL Cell Line Service GmbH (Eppelheim, Germany). Copolymers were synthesized and self-assembled to encapsulate active substance in aqueous solutions [23,24] or conjugated with FA and LA [25] according to previously described procedures.

### 2.2. Characterization

Active substance permeation through Strat-M Membrane (Transdermal Diffusion Test Model, 25 mm, Aldrich, Poznań, Poland) was performed in a PBS environment using Franz diffusion cells (Teledyne Hanson Research, Phoenix DB-6, Variel Ave, Chatsworth, CA, USA), whereas their released amount was evaluated by ultraviolet-visible light spectroscopy (UV–Vis, Thermo Fisher Scientific Evolution 300, Waltham, MA, USA). The absorbance of the formazan product created during the MTT test was read at 570 nm using a microplate reader (Epoch; BioTek, Winooski, VT, USA). Cytometric analyses were performed using an Aria III flow cytometer (Becton Dickinson; Franklin Lakes, NJ, USA) with an FITC configuration (488 nm excitation; emission: LP mirror 503, BP filter 530/30) or a PE configuration (547 nm excitation; emission: 585 nm); at least 10,000 cells were counted. Automated cell confluence analysis, cell density, and viability monitoring were carried out using Live Cell Analyser, JuLI™ Br (NanoEnTek Inc., Seoul, Korea).

### 2.3. Permeation Tests in Franz Diffusion Cells

A PBS solution (15 mL) was introduced into a diffusion cell (acceptor chamber), equipped with a magnetic stirrer. The membrane and donor chamber were placed, and 1.8 mL of the test carrier solution (1.0 mg/mL) was introduced into the donor chamber. The experiment was carried out at 37 °C with constant stirring (V = 400 TPM) for 24 h. During analysis, 200 µL of the solution was taken from the acceptor chamber at specified intervals, and the chamber was resupplied with the same amount of PBS. The samples were analyzed by UV–Vis. Flow-through the membrane (J) was calculated using the following equations:

(1) $$\mathrm{HLB}=20\;\times \frac{M_{\mathrm{hphil}.}}{M_{n}}$$

(2) $$D=\frac{e^{2}}{6\;\times \;t}\;\left(\frac{{\mathrm{cm}}^{2}}{h}\right)\;$$

(3) $$\;J=\frac{D\;\times \;\mathrm{HLB}\;\times \;\Delta c}{e}\;\left(\frac{\frac{µg}{{\mathrm{cm}}^{2}}}{h}\right)$$

where HLB—hydrophilic/lipophilic balance, M_hphil_—molecular weight of the hydrophilic fraction, M_n_—molecular weight of the copolymer, D—diffusion coefficient, e—membrane thickness, t—lag time, J—flow through the membrane, Δc—concentration difference on either side of the membrane [28].

### 2.4. Cell Culture

All cells (Me45, 451-Lu, NHDF, HaCaT) were grown in sterile culture bottles with a culture area of 75 cm^2^ in a DMEM-F12 medium supplemented with 10% (*v/v*) inactivated fetal bovine serum (FBS) (EURx, Gdansk, Poland) and 1% antibiotics (10,000 μg/mL of streptomycin and 10,000 units/mL of penicillin; Sigma-Aldrich, Munich, Germany) at 37 °C in a humidified atmosphere with 5% CO_2_. Cell lines were seeded in a 96-well plate with densities of 10,000 cells per well for MTT tests, and 100,000 cells per well for apoptosis and cell cycle analysis (6-well plate).

### 2.5. MTT Cytotoxicity Assay

For MTT assays, the cells were plated 24 h before drug treatment into 96-well plates at 10,000 cells/well in 0.2 mL of medium. The maximal cell confluence before MTT tests was approximately 70% for NHDF, 100% for HaCaT, 85% for Me45, and 30% for 451-Lu. Appropriate controls, DMSO in the fresh medium were prepared. A series of suspension dilutions (1.563–100 µg/mL for both micelles and conjugates) was added to the wells. The cells were incubated with compounds for 72 h, and the solutions were subsequently removed and incubated with an MTT solution (50 μL of 0.5 mg/mL in RPMI 1640 without phenol red) for 2–3 h. After removing the MTT solution, the formazan crystals were dissolved in 75 μL of an isopropanol:HCl (*v/v* 1:0.04) mixture. The formazan product absorbance was read at 570 nm using a microplate reader. Experiments were conducted triplicate with six technical repeats for each tested concentration, and the results were expressed as the survival fraction (%) of the control.

### 2.6. Apoptosis and Cell Cycle Analysis by Flow Cytometry

An Annexin-V apoptosis assay and propidium iodide (PI) solution (100 μg/mL) uptake test was used to determine the fraction of dead cells after treatment with the particular compound for 72 h. Cells collected from the plates after centrifugation (3 min, 0.6 g, RT) and supernatant removal were suspended in 50 μL of cold Annexin-V labeling buffer and stained with FITF-labeled Annexin-V antibody for 20 min (2.5 μL). The cells were incubated for 20 min in the dark with 10 μL of PI. Annexin-V labeling buffer (250 μL) was added, and the samples were incubated in the dark on ice for 15 min. Cytometric analyses were performed immediately using an Aria III flow cytometer, and at least 10,000 cells were counted. For the cell cycle analysis, the cells were stained with 250 μL of hypotonic buffer (which comprised PI 100 μg/mL in PBS; 5 mg/L of citric acid; 1:9 Triton-X solution; RNase 100 μg/mL in PBS from Sigma, Poznan, Poland), and DNA levels were assessed by fluorescence measurements via BD FACSAria^TM^ III sorter (Becton, Dickinson and Company, Franklin Lakes, NJ, USA) using a PE configuration (547 nm excitation laser line; emission: 585 nm).

### 2.7. Cell Senescence Test

The cells were plated 24 h before drug treatment at 10,000 cells/well in 2 mL of medium. The growth medium was aspirated from the cells and replaced with a medium containing 100 µg/mL of the tested compounds for the next 72 h. After standard incubation, the cells were washed twice with 1 mL portions of PBS per well. The entire wash solution was carefully removed by aspiration to prevent detachment of the cells. A 1.5 mL aliquot of Fixation Buffer (prepared according to the producer protocol, Sigma, Poznan, Poland) was added to each well and the plate incubated for 6–7 min at room temperature. After that, the cells were rinsed three times with 1 mL portions of PBS and then with 1 mL of the staining mixture was added. The cells were incubated at 37 °C without CO_2_ until they were stained blue (24 h). The cells were observed under a microscope; the blue-stained cells and the total number of cells were counted. The percentage of cells expressing β-galactosidase (senescent cells) was calculated.

## 3. Results

**Figure 1:**
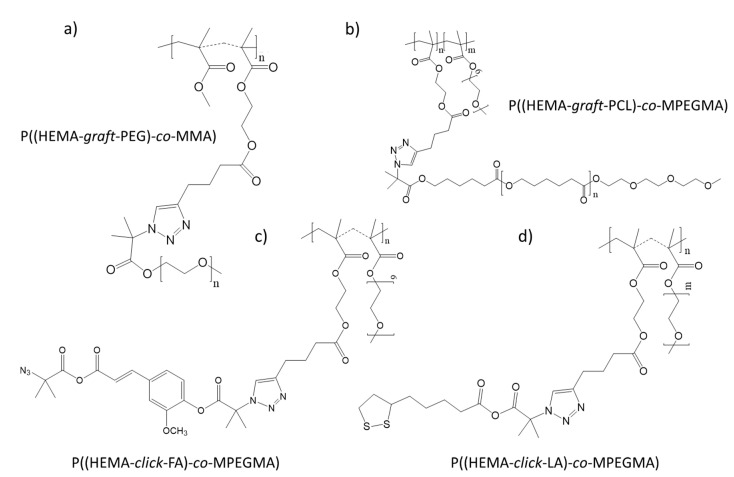
Structures of poly(ethylene glycol) (PEG) graft copolymer (**a**), PEG/poly(ε-caprolactone (PCL) co-graft copolymer (**b**) and conjugates of PEG graft copolymer (**c**,**d**).

**Figure 2:**
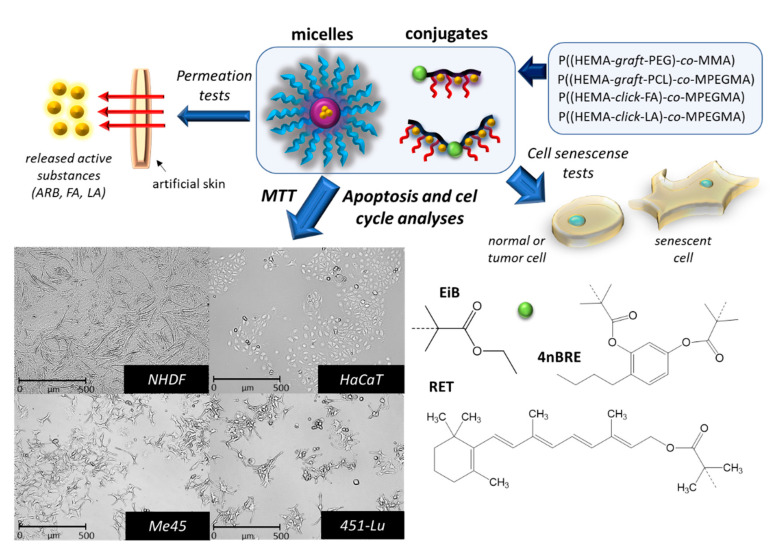
Preliminary biological evaluation of the potential for using carriers in cosmetic products.

The amphiphilic graft copolymers used to prepare the two carrier types, i.e., micelles and conjugates, were obtained from a two-step synthesis combining controlled atom transfer radical polymerization (ATRP) and Cu(I) catalyzed 1,3-dipolar azide-alkyne cycloaddition (CuAAC, “click” chemistry reaction). ATRP was performed using a standard initiator, ethyl 2-bromoisobutyrate (EiBBr), and “bio”initiators, i.e., bromoester derivatives of retinol (RETBr) or 4-*n*-butylresorcinol (4nBREBr_2_). The resulting copolymers of alkyne-functionalized 2-hydroxyethyl methacrylate (AlHEMA) and methyl methacrylate (MMA) or poly(ethylene glycol) methyl ether methacrylate (MPEGMA), i.e., P(AlHEMA-*co*-MPEGMA) and P(AlHEMA-*co*-MMA), were subjected to a “click” reaction with azide functionalized polymers, i.e., biodegradable poly(ε-caprolactone) (PCL), hydrophilic poly(ethylene glycol) (PEG) or azide functionalized low-molecular weight active substances, such as FA and LA. PEG graft copolymers P((HEMA-*graft*-PEG)-*co*-MMA) [23] and P((HEMA-*graft*-PCL)-*co*-MPEGMA) [24] for micellar systems loaded with the active substance, i.e., ARB, and the conjugates varied with the “click” active substance such as P((HEMA-*click*-FA)-*co*-MPEGMA) and P((HEMA-*click*-LA)-*co*-MPEGMA) [25] were synthesized (Figure 1). The active substances are classified as antioxidants and protect against UV radiation, reduce wrinkles and skin discoloration. The most beneficial PEG graft copolymer systems for cosmetic applications based on their physicochemical characteristics, structural parameters (Table 1), delivery properties related to the drug content and the kinetic release profiles (Table 2) were selected for the current biological investigations (Figure 2).

The selected micellar carriers I and II were characterized by high ARB encapsulation efficiencies (77–99%) that were released almost completely within 1.5 h, whereas the content of the chemically bound active substances in the selected conjugates (III, IV) ranged from 30–40%, and then were released in varying amounts within 0.5–4 h (Table 2). The self-assembly ability of the micellar carriers was determined by the critical micelle concentration (CMC), which was lower for heterografted copolymer II due to hydrophobic PCL side chains that corresponded to smaller self-assembled superstructure sizes than for PEG graft copolymer I. However, the polymer conjugates represented smaller particles, in comparison to the micellar systems.

### 3.1. Permeation Tests

Franz chambers, a popular method for measuring the diffusion rates, were used to determine substance permeation released from the carrier through a membrane imitating artificial skin in a PBS environment [29]. The Franz diffusion cell test enabled the in vitro evaluation of polymeric enteric nanoparticles as the dermal carriers with pH-dependent targeting potential [30], or the assessment of the reverse micelle of lipophile-functionalized PEG dendrimer hybrids for applications in carrier-mediated transdermal drug delivery [31]. Therefore, Franz diffusion cells were also selected to evaluate the permeation through the membrane of the studied micellar carriers and conjugates. This study indicated that the active substances were released with efficiencies from 48–73% (^FC^R_max_), depending on the carrier type. These values were lower than those obtained in previous release experiments in PBS for the same polymer systems using a cellulose dialysis membrane MWCO = 3.5 kDa (R_max_ vs. ^FC^R_max_, Table 2, Table 3). This discrepancy may be due to the variety of techniques used to determine the diffusion process for the studied systems by different membrane types. The cellulose membrane release was a preliminary test to determine the release behavior, whereas tests using Franz cells with a membrane imitating skin properties seem to be more reliable in the aspect of potential applications in cosmetology, where the transdermal diffusion plays a crucial role.

**Figure 3:**
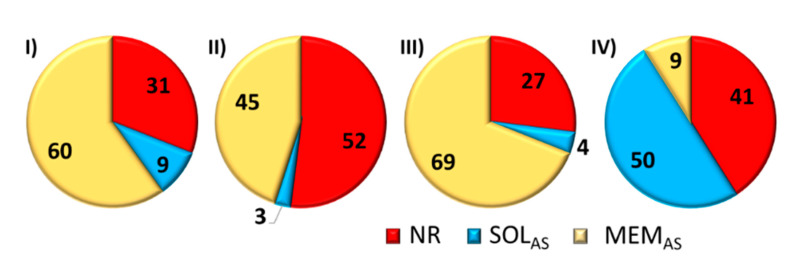
Graph of percentage amount of substance not released (NR), a substance released that passed through the membrane into solution (SOL_AS_), and amount of substance released that remained on the membrane (MEM_AS_).

Franz diffusion cell studies demonstrated the active substance released from carriers I-III barely penetrated the membrane mimicking skin (3–9%) and the rest did not diffuse into solution within 1–4 h (Figure 3). This effect is likely due to the large hydrodynamic diameters of these carriers (I—III), which ranged from 200–420 nm. This implies that micellar systems containing an active substance cannot pass through the skin, but it is possible for the active substance. Thus, a carrier with an unreleased active substance might settle on the membrane surface (skin) and continue to release the substance. The influence of the carrier size on the diffusion process through the membrane was confirmed by carrier IV, which had the smallest hydrodynamic particle diameter (82 nm), and 50% of the released substance diffused into solution (Figure 3). Despite the particle size limitations, the intended use of the described carriers is envisioned, especially in the form of masks or eye pads. In such cases, the carrier can be immobilized on a material or a specially fabricated polymer membrane, where it does not penetrate the skin, but only contacts its surface to release the active substance deep into the skin. Comparing micellar carriers with the same encapsulated substance (ARB) (I vs. II), but differing in their hydrophilic/lipophilic balance (HLB), the length and number of hydrophilic PEG grafts statistically distributed within the backbone, and the extra amount of biodegradable PCL co-grafts in the system II, it was observed that larger amounts of ARB were released into solution by carrier I and it remained the larger amount of released substance in the membrane. Thus, the release of hydrophilic ARB was more efficient from micelles formed by the PEG graft copolymer with a slightly lower content of hydrophilic fraction and a lower HLB, but over a longer time frame (3 h vs. 1 h). For conjugates III and IV, the hydrophobic active substance (FA or LA) covalently bound to the copolymer matrix was more readily released by the conjugate IV as a more hydrophilic carrier with higher HLB levels (Table 3, Figure 3).

**Figure 4:**
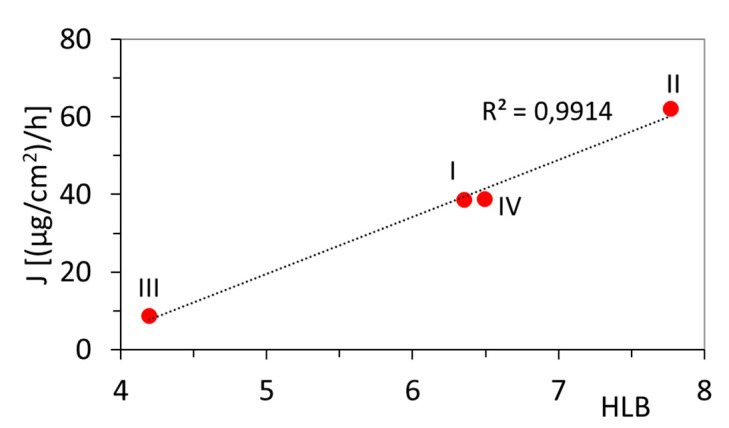
The dependence of the hydrophilic/lipophilic balance (HLB) in graft copolymers on the flow rate through the membrane (J).

The flow rate through the membrane (J), which defines the kinetic efficiency of diffused substance, increased with increasing HLB that represents the hydrophilic fraction in the graft copolymer forming the carrier (Figure 4). Therefore, the lowest flow rate through the membrane, which corresponded to the longest release time (4 h), was obtained for III, which had the lowest HLB coefficient (4.19). However, for II and IV with HLB ≥ 6.5, they were much more functional systems in terms of the flow rate, and they supported high percentage amount of unreleased substance (NR = 40–50%).

### 3.2. Cytotoxicity

**Figure 5:**
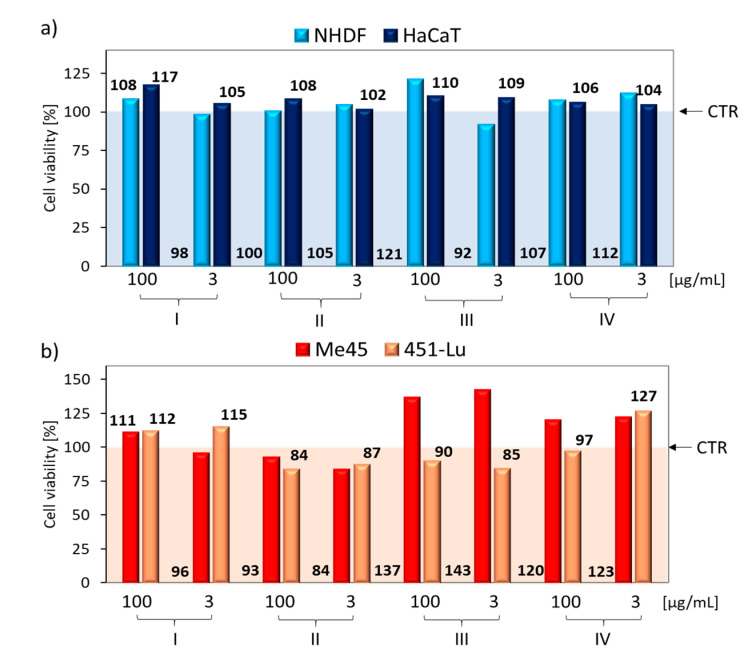
Comparison of in vitro cytotoxicity effects of carriers at higher (100 µg/mL) and lower (3 µg/mL) concentrations in exposure to (**a**) healthy cell lines, and (**b**) melanoma cell lines, where CTR—the control sample viability set to 100% as a baseline for cell viability calculations with the carrier.

The cytotoxicity has been previously assessed for retinoic hydroxamic acid nanoparticles, that showed promise in treating malignant melanoma tumors with high efficacy and low toxicity [32], as well as for a novel mixed polymeric micelles for the co-delivery of paclitaxel and retinoic acid [33]. Moreover, no cytotoxicity of micelles formed by similar copolymers, e.g., brush shaped polyacrylate-*b*-PEG-*b*-polyacrylate or copolymers of poly(2-methacryloyloxyethyl phosphorylcholine) modified PCL have been reported [34,35]. In the present studies, the cytotoxic activity of the synthesized carriers was investigated using two healthy skin cell lines, i.e., normal human dermal fibroblasts (NHDF), human epidermal keratinocytes (HaCaT), and two skin melanoma cell lines, i.e., malignant melanoma cells (Me45) and human metastatic melanoma variant of WM164 cell line (451-Lu). The viability of cells evaluated by MTT assay, which is based on the reduction of the bromide salt of tetrazolium dye to insoluble formazan with a purple color [36,37], was performed at various solution concentrations of carriers with active substances (100–1.5 µg/mL). The MTT test was accomplished after 72 h of cells incubation with carrier solutions and allowed a toxicity assessment of the carriers even after prolonged contact with skin cells. Studies have shown, that micellar carriers (I, II) and conjugates (III, IV) do not affect the viability of healthy skin cell lines (NHDF, HaCaT) (Figure 5a, Figure S1a,b). Cell viabilities obtained at the highest (100 µg/mL) and lowest (3 µg/mL) concentrations were compared (Figure 5). The application of a higher concentrations of the carrier on healthy cell lines, in most cases, resulted in slightly higher cell viability compared to the lower dose (108% vs. 98%, 117% vs. 105%). Thus, in our studies, the active substances released (ARB, FA, or LA) promoted cell viability and proliferation; these may be beneficial for anti-wrinkle cosmetics. The literature data also indicated that topical treatment of tissue with ARB decreased melanin synthesis without affecting cell viability [38]. The obtained results have been also confirmed by published other MTT studies that clearly showed no significant decrease in observed cell viability, when skin cell lines were treated with different ARB [38], FA [39], or LA [40] systems.

Additional MTT tests were conducted on two cancer skin cell lines (Figure 5b, Figure S1c,d). The application of ARB or FA to skin cancer lines inhibited the viability of those lines, according to previously published studies [41,42]. We observed no carrier concentration effect on skin cell tumor viability (Figure 5b). Moreover, in the case of ARB micellar carriers (I, II), a slight increase in cell viability (111/112 vs. 93/84) was demonstrated by carrier I, with higher levels of encapsulated ARB (DLC_I_/DLC_II_ = 99%/77%). This means, in the case of skin melanoma cells, the resulting carriers will not be therapeutic. A similar relationship was detected for the conjugate with FA (III) or LA (IV). They presented the effect of increasing cell viability, especially for the Me45 cell line (cell proliferation 123–143%). However, from the point of view of the target application of the tested carriers with active cosmetic substances, their therapeutic effect on skin melanoma cells was rather unexpected, but it was tested comparatively due to a possible positive therapeutic “side effect”, which in our case did not exist.

**Figure 6:**
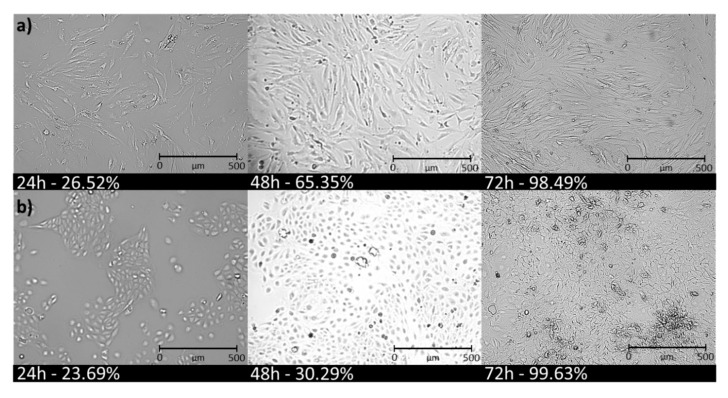
Increase in confluency of (**a**) normal human dermal fibroblasts (NHDF), (**b**) human epidermal keratinocyte (HaCaT) cells treated with PEG graft copolymer-based conjugate III (c = 100 µg/mL).

Exposure of NHDF and HaCaT cell lines to carriers caused a significant increase in cell confluence after a 72 h incubation as compared to control cells (CTR, Table 4). The type of compound used did not affect the confluence level for the HaCaT cell line; however, for the NHDF cell line, the highest confluence was obtained for the FA conjugate (~100%) and the lowest for the LA conjugate (~65%). The concentration (100 µg/mL vs. 3 µg/mL) did not affect cell confluence and additionally confirmed the non-toxicity of these carriers. Under the influence of the used PEG graft polymers, cells do not die and can proliferate. Clear increases in cell confluences after 24 h, 48 h, and 72 h are shown in Figure 6. The confluence of NHDF cells increased by approx. 30% after each subsequent 24 h of incubation, while HaCaT cells initially multiplied more slowly, and then there was a rapid increase in proliferation, which may have been caused by the release of a high dose of the active substance by the carrier. Cell confluence in tumor lines was also observed (Table S1, Figure S2). Me45 cell confluence was detected at approximately 85% in each case, while the 451-Lu cell confluence range was between 26–37%. The higher confluence of Me45 cells compared to 451-Lu is also associated with the obtained MTT results, where much higher viability of Me45 cells was observed (84–143% vs. 84–127%). This means that for the 451-Lu cell line, the action of the compounds does not cause cell death, but their proliferation ability decreased.

### 3.3. Cell Cycle Analysis

**Figure 7:**
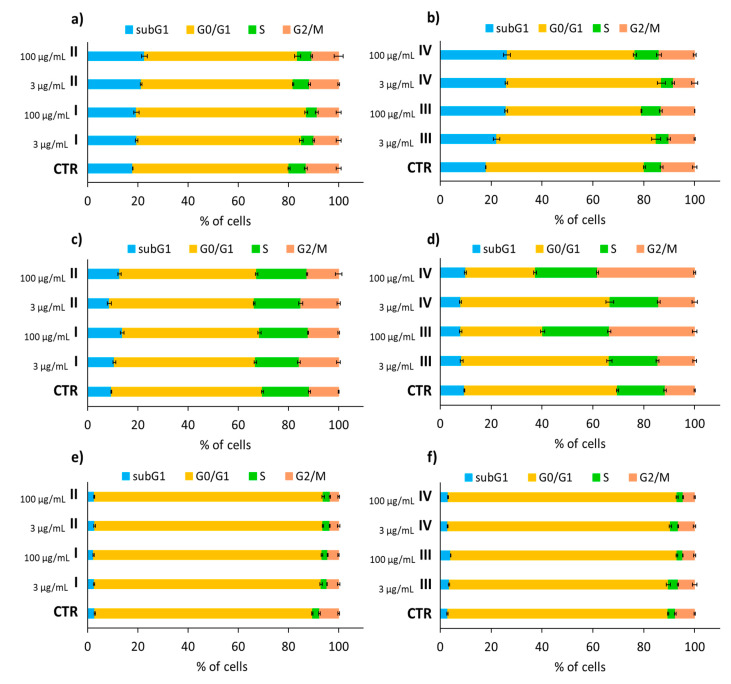
NHDF (**a**,**b**), HaCaT (**c**,**d**), and Me45 (**e**,**f**) cell cycles after carrier solution application at concentrations of 3 µg/mL or 100 µg/mL, followed by a 72 h incubation and compared to untreated control cells (CTR), where subG1—dead cells, G0/G1—mononuclear cells, S—DNA replication, G2/M—mitosis. Means ± S.D. from three independent experiments.

An in vitro evaluation of NHDF, HaCaT, and Me45 model cell lines showed an almost non-toxic effect on human dermal fibroblasts and epidermis, as well as melanoma cells. A cytometric evaluation revealed the effect of the tested carriers with active substances (micelles and conjugates) did not significantly alter the cell cycle phase distribution compared to the untreated control populations of NHDF (Figure 7a,b) or HaCaT cells (Figure 7c,d). NHDF cells were more sensitive to conjugates (III, IV) than to micelles (I, II), and showed a slightly elevated sub-G1 cellular fraction following treatment for 72 h (Figure 7b), regardless of the applied concentration (3 µg/mL vs. 100 µg/mL). However, this increase was insignificant because it was not above 10% compared with control cells. Higher concentrations of the conjugate solution applied to HaCaT cells promoted cell division, which was indicated by a large proportion of cells whose DNA levels doubled (in the G2/M phase) (Figure 7d, Table S2). Cell cycle studies were also performed comparatively on one cancer cell line—Me45. Melanoma Me45 cells were arrested in the G0/G1 phase, which may suggest the cytostatic potential of the active substances released from carriers (Figure 7e,f, Table S3). Although MTT tests did not show a decrease in Me45 cell viability, the use of a micelle solution or conjugate with an active substance may cause arrested the cell cycle and slowed down cell proliferation, which is desirable from a cancer therapy point of view.

### 3.4. Analysis of Apoptotic and Necrotic Changes

Analysis of the sub-G1 cell fraction indicated the presence of a small number of damaged cells containing fragmented DNA but little additional information. Therefore, an apoptosis test was conducted to characterize the type of cell death induced by a carrier solution with active substances. Studies on necrotic (A−/PI+) and apoptotic (A+/PI−, A+/PI+) cell changes provide important information on the cytotoxicity of tested carriers with active substances. The test was based on the use of Annexin-V (that detects early apoptosis) in combination with propidium iodide (PI), which penetrates cells being in necrosis. The cell lines were treated with carriers in two doses: 3 µg/mL and 100 μg/mL.

**Figure 8:**
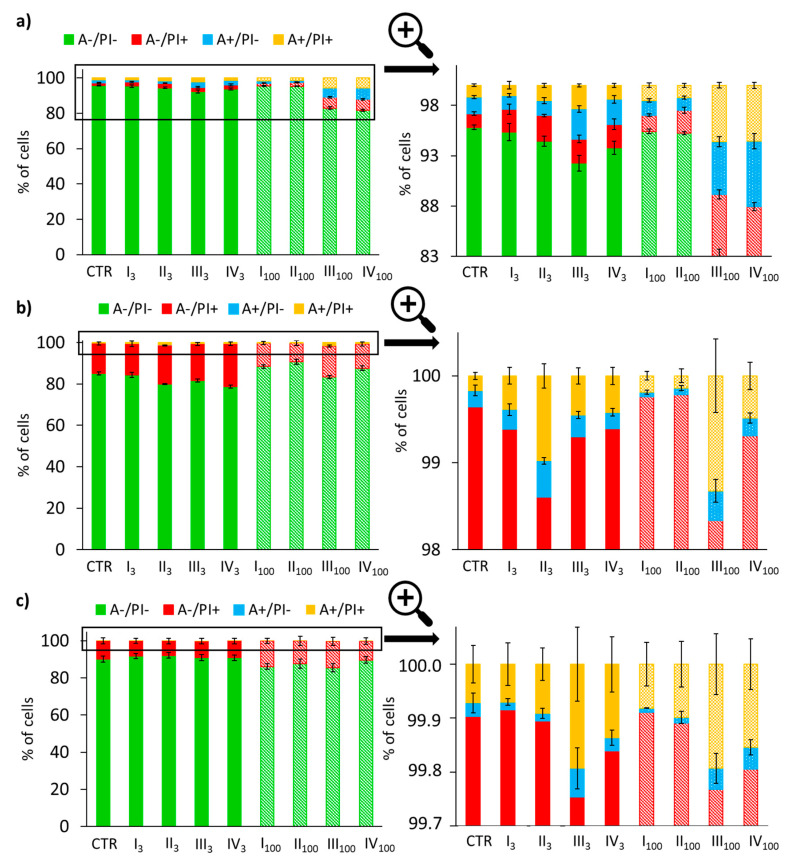
Results of Annexin V/PI double staining apoptosis assay in NHDF (**a**), HaCaT (**b**), and Me45 (**c**) cells after carrier solution additions at concentrations of 3 µg/mL and 100 μg/mL, followed by 72 h of incubation, where A−/PI−: live cells; A−/PI+: necrosis; A+/PI−: early apoptosis; A+/PI+: late apoptosis; 3—3 µg/mL, 100—100 µg/mL. Means ± S.D. from three independent experiments.

The results for the NHDF cells (Figure 8a, Table S4) showed little increase in the necrotic state (A−/PI+ = 2.3–2.6%) or apoptotic state compared with the control (A−/PI+ = 1.4%) for cells treated with a lower concentration of all carrier types (3 μg/mL), and cells treated with a higher micelle concentration (I_100_, II_100_). The slight increase in necrotic (A−/PI+ = 5–6%) and apoptotic (A+/PI- and A+/PI+ = 5–6%) cells detected in samples treated with 100 μg/mL conjugate solutions (III_100_, IV_100_) was small enough that there was no coverage in previously obtained cell cycles or cytotoxicity results. Additionally, there was no significant effect of the carrier or its concentration on apoptosis and necrosis for HaCaT cells noticed in comparison with the control (Figure 8b, Table S5). As in the case of MTT assays, a slightly higher proportion of live cells was observed for higher carrier concentrations (A−/PI−: 79−85% vs. 83−91%). A reverse relationship was observed in the Me45 cancer cell line (Figure 8c, Table S6), where a higher concentration of the carrier resulted in a slight decrease in the number of live cells (A−/PI−: 90−92% vs. 85−90%). Similar to NHDF and HaCaT, necrotic cells for the Me45 line comprised the largest fraction of dead cells, whereas the total number in early or late phase apoptosis ranged in from 0.1–0.3%. In cases with an increase number of cells observed in late apoptosis compared to the control (NHDF: III_100_, IV_100_; HaCaT: II_3_, III_100_; Me45: III_3_, IV_3_, III_100_, IV_100_), the possibility of uncontrolled cell death as a result of mechanical damage was lower, and instead, these cells entered the apoptosis pathway.

### 3.5. Cell Senescence Tests

**Figure 9:**
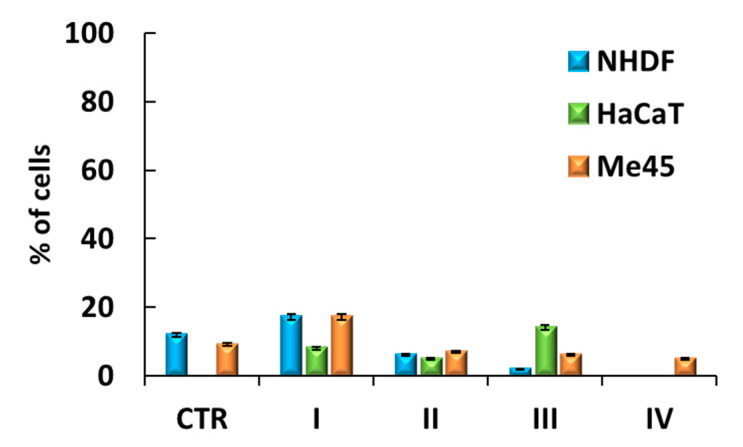
Senescence in cells after a 72 h incubation with a solution containing system III (100 µg/mL).

The last stage for determining the safe use of the tested carriers in cosmetic products was realized by cell senescence tests. Cell aging is associated with the loss of the ability of cells to divide, changes in cell morphology, shape, physical appearance, and gene expression patterns. The assay performed was based on a histochemical stain for β-galactosidase as the most widely used biomarker of cellular senescence activated at pH = 6. Under these conditions, β-galactosidase catalyzes the hydrolysis of the chromogenic substrate, i.e., galactose linked to a substituted indole, which after cleavage gives blue staining easily detectable in senescent cells, but undetectable in quiescent or immortal cells. The senescent cells were observed as blue-stained cells (Figure S3). The share of senescent cells did not increase after treatment compared to NHDF and Me45 control cells; however, for NHDF calls, a slight decrease in the number of senescent cells was detected (Figure 9). For HaCaT cell line, incubation of cells with ARB and FA carriers increased the number of senescent cells, but this effect was small enough to be considered safe.

## 4. Conclusions

Polymeric micelles with ARB loaded by amphiphilic graft copolymers P((HEMA-*graft*-PEG)-*co*-MMA) and P(HEMA-*graft*-PCL)-*co*-MPEGMA), as well as the graft copolymer based conjugates with covalently attached antioxidants P(HEMA-*click*-FA)-*co*-MPEGMA) and P((HEMA-*click*-LA)-*co*-MPEGMA) were designed for delivery of substances applicable in cosmetology, including dermatological problems. The biochemical evaluation of both types of polymer carriers (micelles and conjugates) indicated diffusion of the released active substance into the solution in Franz cells, but its significant part was remaining in the membrane working as the artificial skin. The permeability properties were dependent on the carrier particle size and strongly related to the hydrophilic-lipophilic balance in the copolymer. The application of a higher concentration of the carrier resulted in slightly higher viability of the healthy skin cell lines (NHDF, HaCaT). The exposure of healthy cell lines to carriers caused a significant increase in cell confluence, especially for the NHDF cell line treated with the conjugate with FA. The carriers did not significantly alter the cell cycle phase distribution, the type of cell death of tested cell lines and did not induce cell senescence. The evaluated PEG graft copolymers are promising safe carriers for the transdermal delivery of active substances, e.g., antioxidants, including skin therapy in cosmetology. In vitro biological studies are an excellent starting point for the next phase of mandatory ex-vivo tests on human skin in lab conditions, dermatological studies with a contact test on hypoallergenic tape, and finally in vivo studies on volunteers with different skin types.

## Figures and Tables

**Table 1:** Characteristics of PEG graft copolymers.

No.	Copolymer	I	AS	DP_n_	DP_PEG_	F_hphil._ (wt %)	Ref.
I	P((HEMA-*graft*-PEG)-*co*-MMA)	RETBr	ARB	89	5	32	[23]
II	P((HEMA-*graft*-PCL_9000_)-*co*-MPEGMA)	RETBr	ARB	173	80	39	[24]
III	P((HEMA-*click*-FA)-*co*-MPEGMA	4nBREBr_2_	FA	198	34	21	[25]
IV	P((HEMA-*click*-LA)-*co*-MPEGMA	EiBBr	LA	163	45	33	

I—initiator, AS—active substance, DP_n_—polymerization degree; DP_PEG_—degree of PEG; F_hphil_—the content of hydrophilic fraction in the copolymer.

**Table 2:** Data for micelles (I, II) and conjugates (III, IV), amount of introduced and release active substance from the carriers.

No.	DLC (%)	DC (%)	R_max_ (%)/T (h)	D_h_ ± SD (nm)	CMC (mg/mL)
I	99	-	94/1.5	420 ± 77	0.0836
II	77	-	99/1.5	280 ± 19	0.0080
III	-	32	49/4.0	204 ± 15	-
IV	-	38	96/0.5	82 ± 20	-

DLC: the content of loaded substance; DC: the amount of conjugated substance; R_max_: the amount of the released substance through the cellulose dialysis membrane; T: the release time, D_h_: the hydrodynamic diameter by intensity determined by DLS; CMC: the critical micelle concentration determined by fluorescence spectrophotometry.

**Table 3:** Data for permeation tests.

No.	DLC_apr_ (%)	DC_apr_ (%)	^FC^R_max_ (%)	NR (%)	T_max_ (h)	$D\;(\frac{cm^{2}}{h})\;\times \;10^{-4}$	HLB	$J\;\left(\frac{\frac{µg}{cm^{2}}}{h}\right)$
I	31	-	69	31	3	6	6.35	38.66
II	40	-	48	52	1	6	7.76	62.12
III	-	11	73	27	4	6	4.19	8.89
IV	-	31	59	41	1	6	6.49	38.98

DLC_apr_—drug loading content in the carrier after permeation test, DC_apr_—drug content in the carrier after permeation test, ^FC^R_max_—the amount of released substance using Franz cells, NR—the amount of not released substance, T_max_—a time of permeation test after which the concentration of the substance in the solution did not increase, D—diffusion coefficient, HLB—hydrophilic/lipophilic balance, J—flow through the membrane.

**Table 4:** Average cell confluence after 72 h.

No.	NHDF (%)			HaCat (%)		
conc. [µg/mL]	0	100	3	0	100	3
CTR	63.38	-	-	77.69	-	-
I		72.06	74.35		98.40	95.76
II		68.65	71.40		94.30	92.30
III		99.42	100.00		90.25	94.39
IV		64.54	65.23		91.77	98.04

## Supplementary Materials

The following are available online at https://www.mdpi.com/1999-4923/12/12/1178/s1, Table S1. Average cell confluence after 72 h. Table S2. NHDF and HaCaT cell cycles after carrier addition at concentrations of 3 µg/mL or 100 µg/mL, followed by a 72 h incubation and compared with untreated controls. Table S3. Me45 cell cycles after carrier addition at concentrations of 3 µg/mL or 100 µg/mL, followed by a 72 h incubation and compared to untreated controls (CTR). Table S4. Results of Annexin V/PI double staining apoptosis assays in NHDF cells. Table S5. Results of Annexin V/PI double staining apoptosis assay in HaCaT cells. Table S6. Results of Annexin V/PI double staining apoptosis assays in Me45 cells. Figure S1. In vitro carrier cytotoxicity effects on (a) NHDF, (b) HaCaT, (c) Me45, (d) 451-Lu. Figure S2. Confluency increases in (a) Me45, (b) 451-Lu cells treated with compound III (c = 100 µg/mL). Figure S3. Microscopic visualization of NHDF cell line after senescence test.
