# Supplementary material for: PEG Graft Polymer Carriers of Antioxidants: In Vitro Evaluation for Transdermal Delivery
Source: Pharmaceutics. 2020 Dec 3;12(12):1178. doi: 10.3390/pharmaceutics12121178 (PMC7761655; doi:10.3390/pharmaceutics12121178)
Supplement: Supplementary file 1 [file pharmaceutics-12-01178-s001.pdf]

pharmaceutics

MDPI

Article

Supplementary Materials: PEG Graft Polymer Carriers of Antioxidants: In Vitro Evaluation for Transdermal Delivery

Justyna Odrobińska, Magdalena Skonieczna, and Dorota Neugebauer

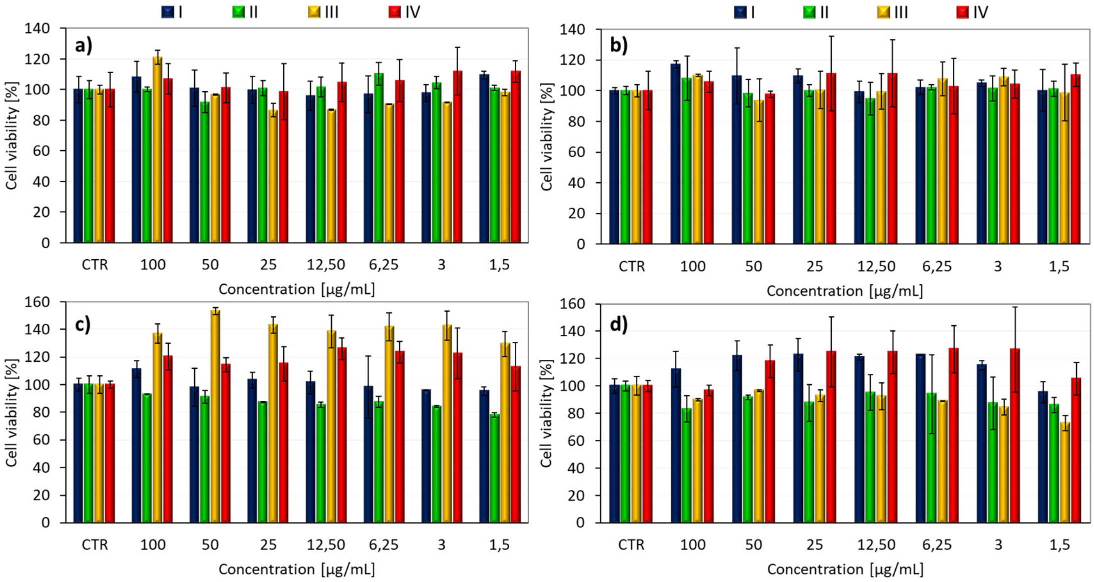

Figure S1. In vitro cytotoxicity effect of carriers on (a) NHDF, (b) HaCaT, (c) Me45, (d) 451-Lu.

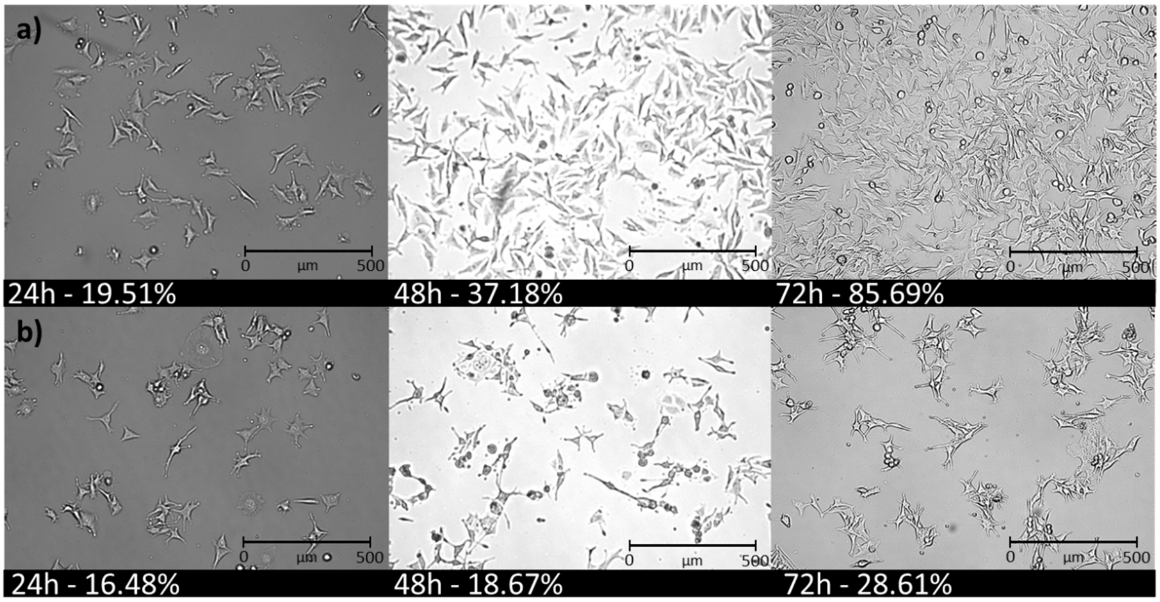

Figure S2. Increase in confluency of (a) Me45, (b) 451-Lu cells treated with compound III (c = 100 µg/mL).

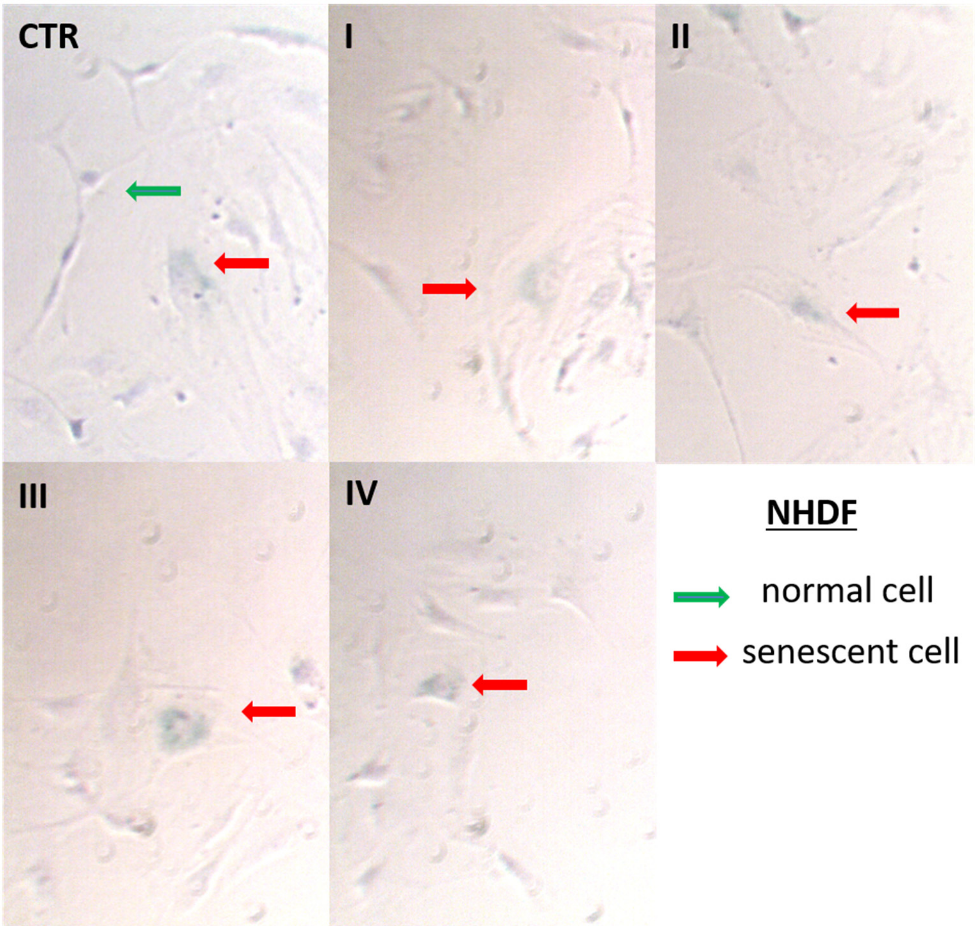

Figure S3. Microscopic visualization of NHDF cell line after senescence test.

Table S1. Average cell confluence after 72h.

	**Me45 (%)**		**451-Lu (%)**	
**conc. [µg/mL]**	**0**	**100**	**0**	**100**
**CTR**	53.26	**-**	25.94	**-**
I		86.70		32.48
II		85.28		37.95
III		85.96		26.04
IV		81.98		33.87

Table S2. NHDF and HaCaT cell cycle after carrier addition followed by 72 h of incubation in comparison to untreated controls (CTR).

	**NHDF (%)**				**HaCaT (%)**			
	**subG1**	**G0/G1**	**S**	**G2/M**	**subG1**	**G0/G1**	**S**	**G2/M**
**CTR**	17.9	62.3	6.7	13.0	9.5	60.2	18.7	11.6
I - 3 µg/mL	19.5	65.7	4.8	9.9	10.7	56.2	17.3	15.8
I - 100 µg/mL	19.4	67.7	4.2	8.7	13.8	54.7	19.4	12.2
II - 3 µg/mL	21.4	60.5	6.5	11.6	8.7	57.7	18.5	15.1
II - 100 µg/mL	22.6	61.0	5.6	10.7	12.6	54.7	20.0	12.6
III - 3 µg/mL	22.2	62.6	5.0	10.2	8.4	58.0	19.0	14.6
III - 100 µg/mL	25.9	53.2	7.6	13.3	8.1	32.2	26.1	33.6
IV - 3 µg/mL	26.0	60.9	4.7	8.4	8.0	58.7	19.2	14.2
IV - 100 µg/mL	26.3	50.2	9.5	14.0	9.9	27.4	24.5	38.2

Table S3. Me45 cell cycle after carrier addition followed by 72 h of incubation in comparison to untreated controls (CTR).

	**Me45 (%)**			
	**subG1**	**G0/G1**	**S**	**G2/M**
**CTR**	2.9	86.7	2.8	7.6
I - 3 μg/mL	2.5	90.5	2.3	4.7
I - 100 μg/mL	2.4	91.1	2.2	4.4
II - 3 μg/mL	2.9	90.9	2.7	3.6
II - 100 μg/mL	2.6	91.2	2.7	3.5
III - 3 μg/mL	3.5	86.1	3.8	6.5
III - 100 μg/mL	4.1	88.9	2.4	4.7
IV - 3 μg/mL	2.9	87.5	3.0	6.5
IV - 100 μg/mL	3.1	90.0	2.3	4.6

Table S4. Results of Annexin V/PI double staining apoptosis assay in NHDF cells.

**NHDF**	**Carrier concentration [μg/mL]**	**% of cells ± S.D.**			
		**A-/PI-**	**A-/PI+**	**A+/PI-**	**A+/PI+**
**CTR**	0	95.82 ± 0.24	1.36 ± 0.18	1.64 ± 0.15	1.17 ± 0.13
**I**	3	95.34 ± 0.85	2.29 ± 0.50	1.37 ± 0.16	0.99 ± 0.37
**II**		94.45 ± 0.49	2.55 ± 0.12	1.47 ± 0.30	1.52 ± 0.21
**III**		92.26 ± 0.79	2.41 ± 0.39	2.99 ± 0.33	2.34 ± 0.14
**IV**		93.77 ± 0.65	2.36 ± 0.53	2.48 ± 0.38	1.39 ± 0.20
**I**	100	95.42 ± 0.24	1.62 ± 0.14	1.45 ± 0.17	1.52 ± 0.23
**II**		95.27 ± 0.16	2.25 ± 0.29	1.24 ± 0.16	1.24 ± 0.20
**III**		82.95 ± 0.74	6.19 ± 0.45	5.26 ± 0.49	5.59 ± 0.27
**IV**		81.78 ± 0.62	6.18 ± 0.39	6.49 ± 0.75	5.55 ± 0.29

A−/PI−: Live cells; A−/PI+: Necrosis; A+/PI−: Early apoptosis; A+/PI+: Late apoptosis.

Table S5. Results of Annexin V/PI double staining apoptosis assay in HaCaT cells.

**HaCaT**	**Carrier concentration [μg/mL]**	**% of cells ± S.D.**			
		**A-/PI-**	**A-/PI+**	**A+/PI-**	**A+/PI+**
**CTR**	0	85.07 ± 0.67	14.58 ± 0.72	0.19 ± 0.06	0.17 ± 0.04
**I**	3	84.34 ± 1.28	15.05 ± 1.30	0.23 ± 0.07	0.39 ± 0.10
**II**		79.91 ± 0.32	18.70 ± 0.18	0.42 ± 0.04	0.98 ± 0.14
**III**		81.74 ± 0.70	17.55 ± 0.71	0.25 ± 0.04	0.45 ± 0.09
**IV**		78.77 ± 0.79	20.62 ± 0.78	0.19 ± 0.04	0.42 ± 0.10
**I**	100	88.50 ± 0.83	11.26 ± 0.80	0.05 ± 0.02	0.19 ± 0.05
**II**		90.66 ± 1.19	9.12 ± 1.13	0.08 ± 0.03	0.14 ± 0.08
**III**		83.40 ± 0.80	14.94 ± 0.44	0.34 ± 0.13	1.32 ± 0.42
**IV**		87.67 ± 1.12	11.64 ± 1.05	0.21 ± 0.06	0.48 ± 0.16

A−/PI−: Live cells; A−/PI+: Necrosis; A+/PI−: Early apoptosis; A+/PI+: Late apoptosis.

Table S6. Results of Annexin V/PI double staining apoptosis assay in Me45 cells.

Me45	Carrier concentration [μg/mL]	% of cells ± S.D.			
		A-/PI-	A-/PI+	A+/PI-	A+/PI+
CTR	0	90.22 ± 1.63	9.68 ± 1.60	0.03 ± 0.02	0.07 ± 0.03
I	3	91.72 ± 1.42	8.20 ± 1.39	0.01 ± 0.01	0.07 ± 0.04
II		92.10 ± 1.51	7.80 ± 1.50	0.01 ± 0.01	0.09 ± 0.03
III		91.04 ± 1.61	8.71 ± 1.52	0.05 ± 0.04	0.19 ± 0.07
IV		90.90 ± 1.54	8.94 ± 1.51	0.02 ± 0.01	0.14 ± 0.05
I	100	86.05 ± 1.49	13.86 ± 1.45	0.01 ± 0.01	0.08 ± 0.04
II		87.60 ± 2.50	12.30 ± 2.48	0.01 ± 0.01	0.10 ± 0.04
III		85.53 ± 2.21	14.23 ± 2.20	0.04 ± 0.03	0.19 ± 0.06
IV		89.68 ± 1.79	10.12 ± 1.78	0.04 ± 0.01	0.15 ± 0.05

A−/PI−: Live cells; A−/PI+: Necrosis; A+/PI−: Early apoptosis; A+/PI+: Late apoptosis.
